# Supplementary figures and images for: Asymmetric-flow field-flow fractionation of prions reveals a strain-specific continuum of quaternary structures with protease resistance developing at a hydrodynamic radius of 15 nm
Source: PLoS Pathog. 2021 Jun 28;17(6):e1009703. doi: 10.1371/journal.ppat.1009703 (PMC8270404; doi:10.1371/journal.ppat.1009703)

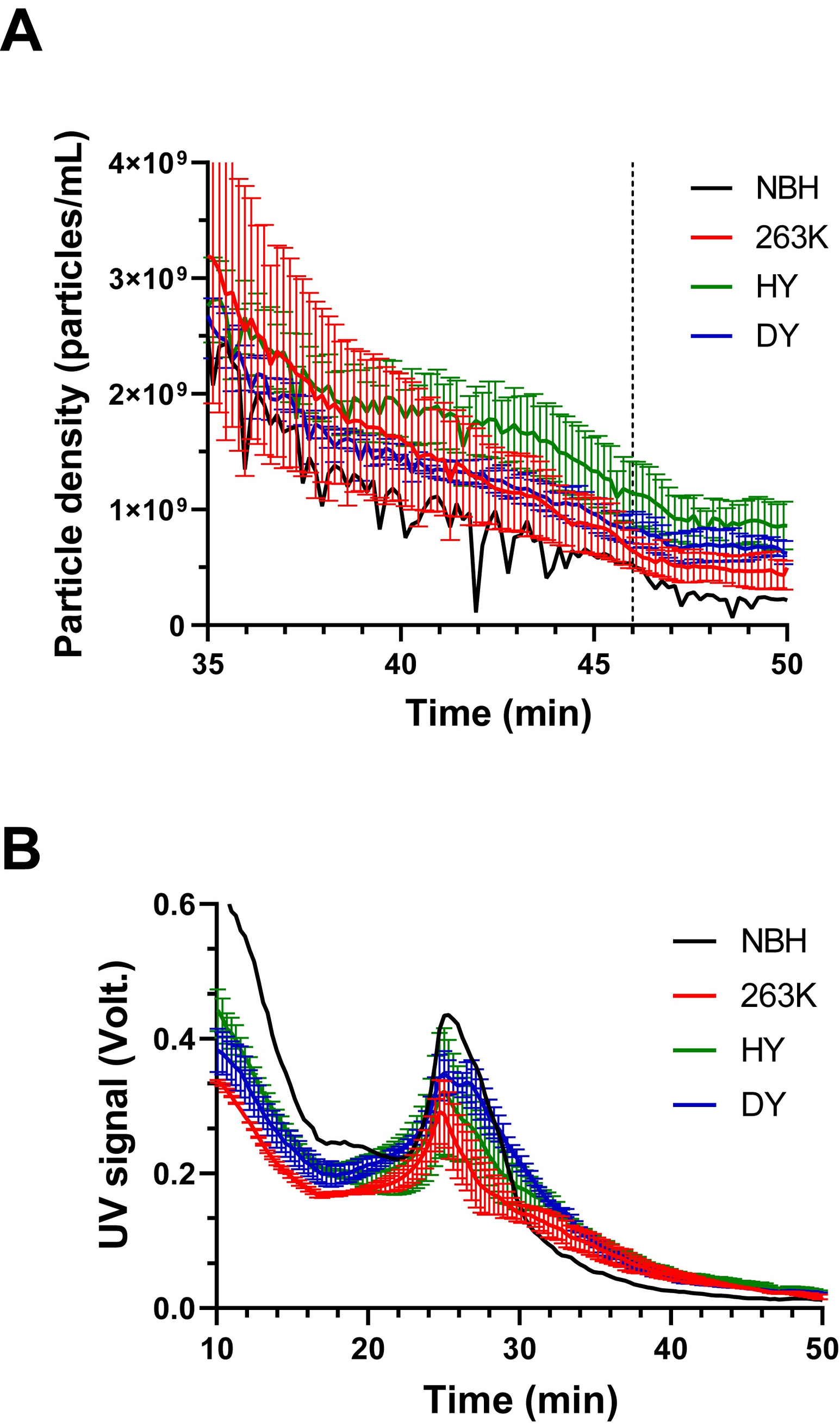

Supplement: S1 Fig — Particle density (A) and UV signal at 280nm (B) for NBH (black), 263K (red), HY (green), and DY (blue) BHs fractionated by AF4. In fractions 46–50, higher particle density and UV signal is evident for prion-infected samples when compared with NBH. (TIF) [file ppat.1009703.s001.tif]

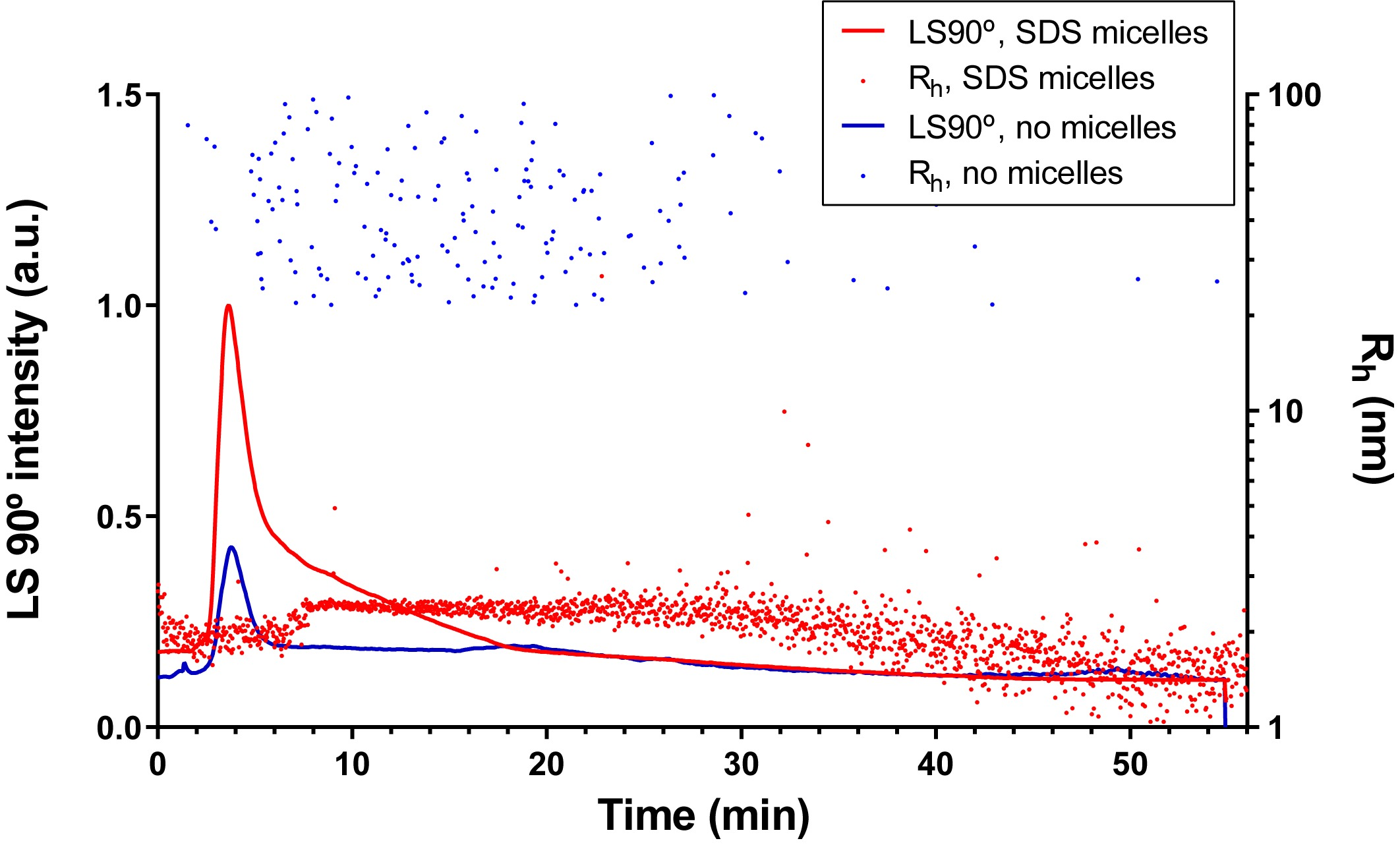

Supplement: S2 Fig — SDS micelles present in the running buffer are evident in the DLS measurements, where background values are ~2–3 nm Rh (red dots). (TIF) [file ppat.1009703.s002.tif]

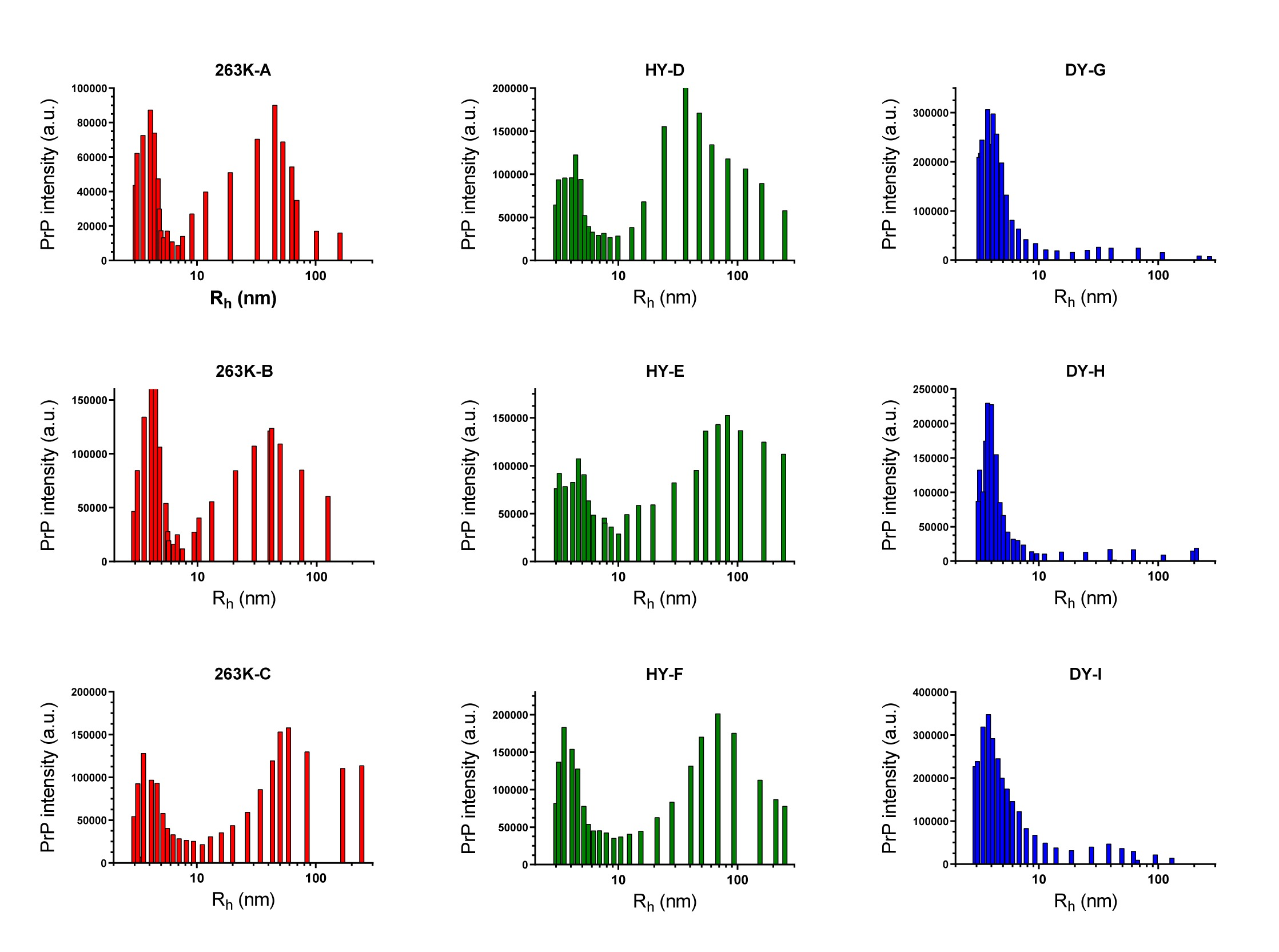

Supplement: S3 Fig — Fifty mM HEPES pH 7.4 containing 150 mM sodium chloride and 0.05% SDS was used as AF4 running buffer. Three brains for 263K (red, A-C), HY (green, D-F), and DY (blue, G-I) were analyzed. (TIF) [file ppat.1009703.s003.tif]
