# Supplementary material for: Asymmetric-flow field-flow fractionation of prions reveals a strain-specific continuum of quaternary structures with protease resistance developing at a hydrodynamic radius of 15 nm
Source: PLoS Pathog. 2021 Jun 28;17(6):e1009703. doi: 10.1371/journal.ppat.1009703 (PMC8270404; doi:10.1371/journal.ppat.1009703)
Supplement: S1 Table — The Rh of particles was calculated from DLS measurements. The length of the particles was calculated fitting the MALS measurements to a rod-shape model. The number of PrP monomers per PrPSc particle was estimated from the PrPSc particle length, assuming a contribution of 1.92 nm in length per PrP monomer and a PrPSc fibril formed by two protofilaments. The PrP normalized intensity was determined by immunoblotting using Sha31 antibody. The relative number of PrPSc particles was calculated by dividing the PrP normalized intensity by number of PrP monomers per PrPSc particle. The maximum possible PrPSc particle density was calculated with ASTRA software (see methods) for fractions 46 and 48, and extrapolated to the rest of the fractions based on the relative number of PrPSc particles. (*) these values of particle density were calculated from MALS measurements directly, fitting the data to a rod-shape model, and assuming a rod radius of 5.5 nm. This calculation was performed for all samples; the specific values obtained for brain E are shown in the table. (DOCX) [file ppat.1009703.s004.docx]

**S1 Table. Particle density estimation for HY assuming a four-rung β-solenoid structure**

| Fraction # | R_h_ (nm) | Length (nm) | PrP monomers per PrP^Sc^ particle | PrP normalized intensity | Relative number of PrP^Sc^ particles | Max. possible PrP^Sc^ particle density (particles/mL) |
| --- | --- | --- | --- | --- | --- | --- |
| 24 | 11.6 | 39.1 | 42 | 0.08316 | 0.00198 | 1.08E+09 |
| 26 | 15.5 | 57.8 | 60 | 0.102213 | 0.001649 | 8.98E+08 |
| 28 | 19.6 | 80.0 | 84 | 0.148777 | 0.001766 | 9.62E+08 |
| 30 | 22.9 | 106.6 | 112 | 0.179116 | 0.001596 | 8.69E+08 |
| 32 | 26.9 | 142.1 | 150 | 0.224708 | 0.001502 | 8.18E+08 |
| 34 | 32.9 | 175.1 | 184 | 0.348884 | 0.001893 | 1.03E+09 |
| 36 | 38.7 | 199.4 | 208 | 0.370763 | 0.001767 | 9.63E+08 |
| 38 | 46.0 | 223.5 | 236 | 0.322006 | 0.001364 | 7.43E+08 |
| 40 | 49.2 | 253.5 | 268 | 0.301998 | 0.001127 | 6.14E+08 |
| 42 | 57.6 | 361.2 | 380 | 0.290421 | 0.000764 | 4.16E+08 |
| 44 | 69.3 | 524.2 | 552 | 0.299313 | 0.000542 | 2.96E+08 |
| 46 | 77.9 | 704.0 | 742 | 0.309167 | 0.000417 | ***2.27E+08* (*)** |
| 48 | 105.6 | 1621.9 | 1708 | 0.394646 | 0.000231 | ***1.26E+08* (*)** |
