# Supplementary material for: Asymmetric-flow field-flow fractionation of prions reveals a strain-specific continuum of quaternary structures with protease resistance developing at a hydrodynamic radius of 15 nm
Source: PLoS Pathog. 2021 Jun 28;17(6):e1009703. doi: 10.1371/journal.ppat.1009703 (PMC8270404; doi:10.1371/journal.ppat.1009703)
Supplement: Attachment — Submitted filename: Response to reviewers_Cortez et al.pdf [file ppat.1009703.s005.pdf]

Centre for Prions and Protein Folding Diseases
204 Brain and Aging Research Building
University of Alberta
Edmonton, Alberta T6G 2M8
Canada

May 25, 2021

Dear Editors:

Thank you for your letter (March 12th, 2021) and comments on our manuscript PPATHOGENS-D-21-00149 - [EMID:5fa37fb8d3adef27] entitled "Asymmetric-flow field-flow fractionation of prions reveals a strain-specific continuum of quaternary structures with protease resistance developing at a hydrodynamic radius of 15 nm". We have now performed additional experiments to revise our manuscript as per your suggestion and have addressed all the reviewer's comments. We herein submit the revised manuscript for your consideration and possible publication in PLOS Pathogens.

Part II – Major Issues: Key Experiments Required for Acceptance

Reviewer #1: 1) Most importantly, although the authors would have no way of knowing this prior to submission, the structure of the 263K PrP^{res} has recently been solved by cryo-EM and the structure is now posted on bioRXiv (https://doi.org/10.1101/2021.02.14.431014). This structure reveals that in contrast to the assumptions made in the current manuscript, the ordered fibril core is a single PIRIBS-based filament (13 x ~4 nm) and not a pair of 4-rung beta-solenoid-based protofilaments. This alters the fundamental assumptions about monomers per unit length that the authors have used for their calculations presented in Table 1 and elsewhere. Thus, these calculations should be redone accordingly. Although I doubt that such recalculations will materially alter the general conclusions of the manuscript, they will certainly make the estimates presented in Table 1 more accurate for 263K.

We agree with reviewer #1 that the recently published high resolution structure of 263K PrP^{res} provides us with a much better model for our calculations. We re-calculated the number of monomers per unit assuming a PrP^{Sc} fibril core of a single PIRIBS-based filament with a contribution 0.49 nm per monomer. Almost double the monomers calculated with the 4RBS model are needed to form fibrils with the same length. We replaced the values in Table 1 (fourth column) and text accordingly. For comparison, we have moved the calculations based on the beta rung solenoid structure to supplemental material.

We also re-calculated the maximum possible PrP^{Sc} particle density (last column in table 1) using an average diameter of 8.5nm (13nm maximum and 4nm minimum diameter of the PIRIBS structure). The new calculation resulted in an increase in the number of particles by 2.9 times.

2) L206 and thereafter: In these calculations, it seems that the lateral bundling of fibrils, and its effects on mass per unit length calculations should also be considered. Measurements of the ratios of radii of hydration and gyration could give indication of how elongated the particles are and, hence, the extent to which the fibrils are bundled laterally.

We agree that this shape information could be more precisely determined with both radius of hydration (R_h) and gyration (R_g). Unfortunately, a precise concentration of the particles giving rise to the measured light scattering signal is required for calculation of R_g. The complexity of our sample means that several different particle types (non-PrP particles) are contributing to the overall light scattering, at least in the small and middle fractions, and we cannot know the exact proportion contributed by PrP. Because the AF4 separates based on R_h, and the fact that we can directly measure R_h during elution, we have confidence that all particles, PrP and others, have the same R_h in a given fraction. However, particles with the same R_h may have differing shapes and therefore different R_g. Only by injecting a highly purified PrP^{Sc} sample, as done in the original Silveira et al Nature paper, could we make use of light scattering measurements to determine R_g in small and medium fractions. Because the premise of our isolation method was to isolate the full range of PrP^{Sc} particles from brain with as little perturbation as possible, the consequence was a sample containing many non-PrP particles.

Despite this, we agree that the concept of lateral bundling is important to highlight, so we have included the sentence "*Although lateral bundling of PrP^{Sc} fibrils might also occur, its effect on mass per unit length calculations was not considered here*" (L214)

Reviewer #2: 1) A major weakness is that the study did not directly address the relationship of various PrP aggregates to prion infectivity. It is understandable that animal bioassay is probably too costly, but authors could consider using cell based infectivity assay to at least show the correlation. Authors used RT-QuIC assay to show the seeding activity. So far, there is no evidence that RT-QuIC product is infectious and there is no study to definitely show a correlation between RT-QuIC positivity and prion infectivity. RT-QuIC seeding activity cannot reflect prion infectivity and pathogenicity.

The reviewer has requested a demonstration that the "seeding activity" we detected in RT-QuIC correlates with "infectivity", as measured by animal or cell assay where true PrP^{Sc} is produced, unlike RT-QuIC where the product itself is not infectious. It is true that a hamster bioassay is beyond the scope of our work, so we have instead used a cell assay and the PMCA amplification assay, both of which produce actual infectious

PrP^{Sc}. We were fortunate to be able to acquire CAD5 cells expressing hamster PrP on a mouse *Prnp* knockout background from Dr Joel Watts (Bourkas et al. *J Biol Chem.* 2019; 294, 4911–4923.). There are very few cells lines susceptible to hamster prions, but Dr Watts has published his success with this cell line for amplifying Hyper prions. Unfortunately, he cautioned us that he was not able to amplify Drowsy in this cell line, so we were not sure if we would be able to succeed with Drowsy fractions, given that they contain much less PrP^{Sc} than initial brain homogenates. Therefore, in parallel, we also attempted PMCA reactions in hamster brain homogenate substrate to test for infectivity in fractions of Hyper and Drowsy, as PMCA also generates *bona fide* PrP^{Sc}, albeit with lower efficiency for Drowsy (Shikiya et al. *J Virol*. 2010;84(11):5706-5714. doi:10.1128/JVI.00243-10).

For the cell culture study, after 7 passages of cell culture (as recommended by the Watts publication), we were delighted to see not only propagation of Hyper PrP^{Sc}, but also Drowsy PrP^{Sc}. While we want to be cautious about commenting too strongly on titre based only on the amount of PrP^{res} seen (as we are not accounting for variables of aggregate stability or clearance in cell culture), we do see the strongest PrP^{res} signal in cultures treated with the larger aggregates for both Hyper and Drowsy, indicating that our fractions do indeed contain infectious prion particles.

With respect to correlating the seeding activity data from RT-QuIC and infectivity data from the cell cultures, we treated cultures with the same volume of fraction, regardless of PrP content, and also repeated our RT-QuIC using equal volumes for each reaction (as opposed to adjusting for PrP content first). The results for these new experiments were included in Fig 7. For Hyper and 263K, adding the same volume of all fractions meant that proportionately much less PrP was added from the smaller fractions. The new RT-QuIC data shows, as expected, that the fractions containing the larger particles were more efficient at seeding than the fractions containing the smaller particles (4-5hr lag phase vs 10hr). This matches nicely with the cell infections, where we see more PrP^{res} signal in cultures seeded with the larger fractions, but still some infectivity in the smaller fractions. For Drowsy, our original RT-QuIC data (normalized to PrP content before seeding), also showed that larger particles were more efficient at seeding than the smaller particles (10hr vs 60hr lag phase). After repeating with equal volumes of fractions, all lag phases were equal (10hr), again indicating that the larger particles are more efficient (because there is much less PrP in the same volume of a larger Drowsy fraction). The cell culture results for Drowsy also indicated that the large aggregates are infectious and appear to be more efficient because we were not able to detect PrP^{res} after infection with the smaller fractions. Of course, given that the majority of Drowsy particles are in fact protease-sensitive, we cannot comment on whether we successfully generated a protease sensitive population of particles in culture, as the readout from cell culture is only the presence of PrP^{res}. Interestingly, the lag phase of 10hr in Hyper smaller fractions, correlated with a lower PrP^{res} signal on immunoblot after 7 passages of culture, and was comparable to the level of PrP^{res} seen for larger Drowsy fractions which also had a 10hr lag phase. However, without full titration and timecourse experiments (or indeed, animal experiments), we are reluctant to overstate this correlation between RT-QuIC lag phase and PrP^{res} level in culture, especially since we

do not know how high PrP^{res} level can even be achieved in cell culture infected with Drowsy, as it has never been done before. It is also possible that more passaging would reveal some infectivity in the smaller fractions.

We also performed PMCA reactions, but were not able to get convincing baseline amplification for Drowsy, even starting with the Drowsy brain homogenate. Our conditions did allow for amplification of Hyper, with relative amounts of PrP^{res} from different sized fractions mirroring what was seen in cell culture (See Figure 1 below). Given that our results from cell culture showed results for a fast and slow strain, we chose to only include the cell culture data, not PMCA data, in the final paper (Figure 7E).

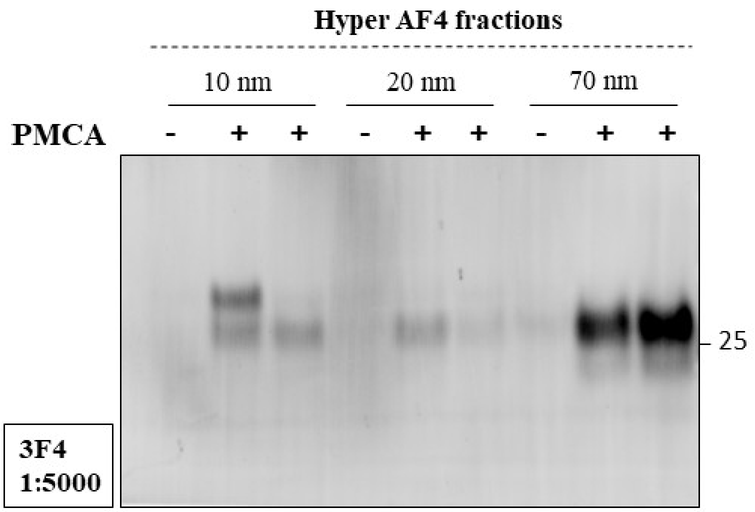

Figure 1. PMCA amplification of Hyper AF4 fractions. *PMCA was performed as described previously (Duque Velasquez C et al., 2020; Johnson CJ et al., 2012) using a Q700 sonicator (Qsonica) connected to a circulating water bath (Thermo Fisher Scientific). Briefly, hamster brain homogenates were centrifuged at 500 x g for 30s. To the supernatant, 3 teflon beads were added and seeded with AF4 fractions of different sizes at 10^{-1} dilution and sonicated for 96 cycles with 30s on and 14:30 s off cycle. Each amplification reaction was done in duplicate and the product of PMCA amplification was analyzed by immunoblotting after PK digestion with 50 µg / mL PK. Non-sonicated samples were incubated at 37°C. While incomplete PK digestion occurred in one of the 10 nm samples, PrP^{res} was detectable in the other sample. Also, low levels of PrP^{res} could be seen in non-amplified 70 nm samples, but the signal was greatly amplified after one round of PMCA.*

We hope the reviewer accepts this PMCA and cell culture data as proof of infectivity within our samples, as requested. We have also added the cell culture data to our paper, including the caveats that we cannot exclude the influence of aggregate stability

and clearance in culture, nor the presence of protease-sensitive particles. We will also continue to word our findings about RT-QuIC results as seeding activity only, as even propagation of PrP^{res} in cell culture does not truly prove pathogenesis in an animal.

Further to the question about correlating RT-QuIC amplification with infectivity, we have identified several references to papers that have in fact shown correlations between RT-QuIC and infectivity, including Wilham et al., PLoS Path. (2010), which showed a striking correlation between prion infectivity and RT-QuIC positivity, and Vascellari S et al., PLoS One 2012, which is particularly relevant as this paper showed that for strains with little detectable PrP^{res}, but lots of infectivity from more protease-sensitive PrP particles, RT-QuIC activity correlated better with infectivity than PrP^{res} (Vascellari S et al., PLoS One 2012).

Lastly, we also acknowledge that the main concern in any amplification assay, such as RT-QuIC, is a false positive. If conditions are not set properly, or if the PrP substrate is of low quality, one can get spontaneous aggregate formation or can seed reactions with non-infectious material. In such a situation it would indeed be incorrect to assume that increasing ThT signal correlated with infectivity. It all depends on the conditions. To demonstrate that our conditions did not result in false positives, we have now included control RT-QuIC data (dotted black line in Fig. 7A, B, and C), in which we seeded the aggregation reactions with fractions from a normal brain homogenate fractionation. No false positives were seen.

2) The RT-QuIC assay showed seeding activity in 10 nm particles of all three strains, which is comparable to the seeding activity in larger particles. Since there is no PK-resistant PrP in these particles (at least for DY strain), some discussion about the possible seed in these fractions would help. This result itself is quite interesting if the same fraction can be tested for prion infectivity assay as mentioned above.

We were not able to amplify small fractions of drowsy prions in PMCA or in cell culture so unfortunately cannot comment on the infectivity of these smaller Drowsy particles, but the smaller HY particles were indeed infectious. An important possibility suggested by reviewer 1 was that the seeding activity we saw in smaller particles was influenced by a “tail” effect of imperfect fractionation, such that increasing the volume of fraction to normalize to PrP content led to extra “tail” or PrP^{res} and might have given more seeding activity than expected. By repeating the experiment using equal volumes for RT-QuIC, PMCA and cell culture, we can conclusively say that we are not seeing a “tail” effect, as the PMCA amplification and PrP^{res} signal in cell culture was lower for the fraction in between the largest and smallest. If a tail effect were present, we should see a gradual diminishing signal as we move farther from the peak in the large fractions.

We have added some prose about why we may not see cell culture PrP^{res} induced by the smaller DY particles, including the potential influence of aggregate stability and clearance on smaller Drowsy particles in culture, allowing them to be degraded or cleared (L312-L340).

3) Could the solubilization step alter the size of PrP aggregates? Is it possible that the less stable DY strain is more vulnerable to the solubilization treatment, which results in more PrP being dissociated from the complex? In that case, various sizes of the particles may actually reflect the braking down products instead of native products in diseased brains.

We agree with reviewer #2 that that the size distribution of PrP particles could be affected by the solubilization of the samples, especially for DY which has less stable PrP aggregates. We do have evidence that we have not completely broken down all the particles because when we fractionate the same solubilized sample under high salt running buffer during fractionation, we see a dramatic loss of larger aggregates in favour of smaller ones. Therefore, while it is impossible with our current study to exclude some effect, we are confident that we have preserved at least some of the larger more vulnerable aggregates. The only way to prove that the size distribution seen is also present in brain, would be to image the aggregates *in situ*. Unfortunately, there is no current method to specifically detect all types of PrP^{Sc} aggregates (PK sensitive and resistant), without the confounding detection of PrP^{C} and without using harsh treatments for epitope retrieval, which may also disrupt fragile aggregates. Instead, we can only acknowledge this caveat and we have added the sentence "*Although we intentionally chose sample solubilization conditions that minimize the dissociation of PrP aggregates, the necessary presence of detergents in the solubilization and AF4 running buffers could still affect the actual size distribution of native PrP particles present in the prion infected brains, especially in the case of DY*" at the end of discussion (L466).

4) The strong PK resistant signal in fractions greater than 15 nm R_h was interpreted as that "This result strongly suggests a strain independent conformational change in PrP^{Sc} structure once particles reach 15 nm R_h." If the sizes of particles actually reflect the breaking down product during solubilization step, this observation could be interpreted in a different way that the PK-resistant conformation cannot be maintained when the particle is smaller than 15 nm R_h.

We agree with this interpretation and think it is very important to include as an alternate possibility. We included the following sentences "*Alternately, if the smallest particles with partial resistance to PK digestion simply came from the fragmentation of larger PrP^{res} particles in the brain or during sample preparation, the 15nm R_h PrP^{res} particles might not reflect the point at which conformational change occurs, but rather the smallest size at which a PK-resistant conformation can be maintained*" (L402). We have also replaced the sentence "*This result strongly suggests a strain independent conformational change in PrP^{Sc} structure once particles reach 15 nm R_h*" with "*This result strongly suggests that PrP^{Sc} particles cannot maintain PK-resistance below 15 nm R_h, regardless of strain*" (L296). We also modified our title to read "Asymmetric-flow field-flow fractionation of prions reveals a strain-specific continuum of quaternary structures with **protease resistance developing** at a hydrodynamic radius of 15 nm."

Reviewer #3: None

Part III – Minor Issues: Editorial and Data Presentation Modifications

Reviewer #1: 3) L130-143: Another possible explanation for the lower ratio of PrP^{res}:total PrP could be that DY has less PrP^{Sc} per unit of tissue, thus decreasing the PrP^{Sc}:PrP^{C} ratio, and therefore the PrP^{res} to total PrP ratio.

It is true that less PrP^{Sc} in the tissue could lower the ratio of PrP^{Sc} to PrP^{C} and therefore the ratio of PrP^{Sc} to total PrP, but our figure 1C shows total PrP is the same across all the strains. Assuming that PrP^{C} levels are the same in the hamsters infected with the three strains, the only way to have equal total PrP levels is by having the same amount of PrP^{Sc} and PrP^{C} added together. We suspect that the lower PrP^{Sc}: total PrP ratio in DY is not from less PrP^{Sc} *per se*, but less PK-resistant PrP^{Sc}. Otherwise it would mean that the hamsters infected with DY actually have more PrP^{C}, to compensate for less PrP^{Sc}, such that the total PrP is the same for all. They would have to have the precise amount more PrP^{C} that they have less PrP^{Sc}, which seems unlikely. Nevertheless, the assumption about all strains having the same levels of PrP^{C} is worth pointing out, given that DY animals are older at end stage. We have added a sentence stating that this calculation is made assuming all animals have comparable levels of PrP^{C} (L144).

4) I cannot find reference to Fig. 2D in the main text.

This figure (now Fig. 2E) is now referenced.

5) L220: criteria should be criterion

The word “criteria” was replaced by “criterion”.

6) L239: It would be helpful to refer to Fig. 2B here.

Fig. 2B is now referenced.

7) L275 and beyond: It is important to point out that this type of analysis only probes the PK sensitivity of the epitope of the antibody used, which is not clear here (to me at least). Other parts of the structure of a given strain may or may not be similarly PK-sensitive.

This is a very good point. We have clarified this section accordingly, with a clearer definition of PrP^{res} as measuring the presence of Sha31 epitope (or loss thereof) during PK digestion, and an assumed loss of the associated resistant core of this region (L284-287). We also included a caveat at the end of the discussion indicating that, because of

this, we cannot comment on the existence of assemblies that might retain some core resistance at locations more C-terminal to this epitope (L469).

8) L284-5 (for example): The wording here ("conformational change in PrP^{Sc} structure") and elsewhere implies that the authors assume that PK-sensitive PrP species that are bigger than PrPC in NBH, but smaller than 15 nm, are a form of PrP^{Sc} (which they define in the beginning as being infectious). What is the evidence that such species are a form of PrP^{Sc}, rather than, say, some non-infectious oligomeric intermediate that might be induced by PrP^{Sc} in the brain, but dissociated in detergent? In that case, a better description would be that PrP^{Sc} particles that are stable under these experimental conditions have an R_h of > ~15 nm.

Agreed. We have redefined PrP^{Sc} in the abstract, introduction and throughout, to make it clear that we describe PrP^{Sc} as a constellation of assemblies that are associated with disease, ranging from small, soluble, protease-sensitive (PrP^{sen}) oligomers, to large, less soluble, partially protease-resistant (PrP^{res}) fibrils. Our goal was to isolate PrP^{Sc} aggregates, defined as all types of PrP aggregates that are specifically associated with prion infection, whether directly infectious themselves and resistant to proteinase K or not. We also rephrased our comments around structural change, to emphasize the possibility that PrP^{Sc} particles cannot maintain PK-resistance below 15 nm R_h, as opposed to declaring that a specific structural change is occurring at that size.

9) L289: Analogous to (7) above, this analysis probes only the stability of the epitope of the antibody used, not the whole particle, and should be described as such.

Agreed. We have revised the text accordingly (L299 and L308). See response to (7) for details.

10) RT-QuIC data, Fig 7: It would be informative to also show the relative seeding activities as a function of size before they are adjusted for PrP concentration. I assume, based on Fig 2C, that roughly 4x more equivalents of the 10 nm fraction would have been used to seed the reactions than the larger particle fractions. Such results might weigh in on the prior question of whether such particles are really some form of PrP^{Sc}. The existence of such particles with seeding activity would be very interesting in their own right, but not if the seeding activity in the 10 nm fraction is simply due to spillover of larger seeds from adjacent fractions during fractionation. In any case, such quantitative comparisons are difficult to make accurately based on RT-QuIC lag phase alone.

As reviewer #2 presumed, in order to seed the reaction with the same amount of PrP, we have used around 3-5 times more volume of fractions containing particles of 10nm R_h than of fractions containing particles of 70nm R_h for HY and 263K. Thus, the high seeding activity observed for the 10nm particles could be the result of a spill over of the larger particles from adjacent fractions. This questions the seeding activity of the 10nm PrP particles. In case of DY, we used 4 times less volume of 10nm than of 70nm particles.

To address this, we have now run an RT-QuIC assay seeding the reaction with the same volume of all fractions. In the case of 263K and HY, we saw a longer lag phase for the 10 and 20nm particles than for the 40 and 70nm particles, keeping open the possibility that the seeding activity of the 10nm particles is due to a spill over of larger fractions from adjacent fractions. To further address this, we performed PMCA and cell culture infections. While we could not amplify Drowsy by PMCA, we were able to amplify Hyper, and saw more PrP^{res} signal in infections with the large fractions (70nm), less with the smallest fraction (10 nm) and least with the middle (20 nm) fraction. If our infectivity was simply due to a tail effect of the larger particles during fractionation, we would have expected decreasing infectivity as we moved to smaller fractions. This was not the case, since the lowest level was in the middle fraction. We also repeated this with cell culture infections, finding comparable findings for Hyper infection. Drowsy also caused infection, but only large particle infection was observed, so we cannot comment on a tail effect directly from these experiments Lastly, we repeated our RT-QuIC assay adding the same volume for all particles sizes. For Drowsy, this resulted in almost the same lag phase for all particles. The 10nm particles are 6 fractions (6 minutes, 1.2mL) apart from the 20nm particles, 14 fractions (14 minutes, 2.8mL) apart from 40nm particles, and 22 fractions (22 minutes, 4.4mL) apart from the 70nm particles. If the seeding activity observed with the fraction containing mostly particles of 10nm would be due to a spill over of the large particles from the adjacent fractions, the lag phase for the 10nm particles should be longer than for the 40 or 70nm particles. All together, we feel these results discount any major tail effect from fractionation. We have included the new RT-QuIC data and cell culture data in the manuscript as Fig. 7, including a description of the findings.

11) L315-316: A thought to consider: This result would be the expected result if HY and 263k were largely single fibrils for which only the ends have seeding activity. In this case large fibrils have the same number of templating surfaces (i.e., 2) as smaller ones. On the other hand, the larger DY particles may either be non-fibrillar assemblies with more than 2 templating surfaces per particle, or bundles of shorter fibrils such that the bigger the particle, the more templating surfaces it has per particle.

This is an interesting possibility. We have now done EM on the fractions and can confirm the presence of fibrils in both HY and DY larger fractions. We have included these images in Fig. 2. While there could be some lateral bundling occurring, the overall structures seen between HY and DY are comparable, so we do not think that a major difference in assembly is driving the difference in seeding ability.

12) L367: Again: how does one know that the PrP^{sen} components have any replication activity (or infectivity?)

The best way to address this was to repeat our experiments with equal volumes of fraction, in RT-QuIC, PMCA and cell culture. The results (described in answer to number 10) ensure we are not seeing a “tail” effect, as the PMCA amplification and PrP^{res} signal in cell culture was lower for the fraction in between the largest and

smallest. If a tail effect were present, we should see a gradual diminishing signal as we move farther from the peak in the large fractions.

13) L434: I think that "the main contributor" is too strong here because this work has not probed or defined secondary or tertiary structures, and, therefore, these structures cannot be assumed to be the same for these strains. In fact, FTIR comparison of these strains provide evidence that the beta sheet secondary structure of DY is significantly different from HY and 263K (ref 40). Thus, "a significant contributor" would be more appropriate here and elsewhere when this conclusion is expressed.

We replaced “the main contributor” by “a significant contributor” or “a major contributor” throughout.

Reviewer #2: 5) Since PrP^{Sc} generally means the PK-resistant and infectious PrP form, the term PrP^{Sc} used in this manuscript should be defined.

We have redefined PrP^{Sc} in the abstract, introduction and throughout, to make it clear that we describe PrP^{Sc} as a constellation of assemblies that are associated with disease, ranging from small, soluble, protease-sensitive (PrP^{sen}) oligomers, to large, less soluble, partially protease-resistant (PrP^{res}) fibrils. We also added a sentence at the beginning of the results section “*Our goal was to isolate PrP^{Sc} aggregates, defined as all types of PrP aggregates that are specifically associated with prion infection, whether directly infectious themselves and resistant to proteinase K or not*” (L129).

Reviewer #3: 1) Are there any EM data showing the presence of fibrillar structures in the different AF4 fractions, particularly those above 15 nm? These data would provide further justification for fitting the light scattering data to a rod-shaped model (lines 378-380). It would also provide support for assuming that fibrillar assemblies are present in fractions which contain larger PrP^{Sc} particles.

We explored the AF4 fractions by EM and were able to document fibrillar structures in both HY and DY strains. We have now included in Fig. 2 fibrillar structures present in fraction #47 (containing particles > 70nm R_h), enriched in larger PrP^{Sc} particles. We also found fibrillar structures in fraction #30 that contain particles of 23nm R_h, or 106nm length as calculated by MALS.

HYPER - Fraction #30 (23 nm R_h):

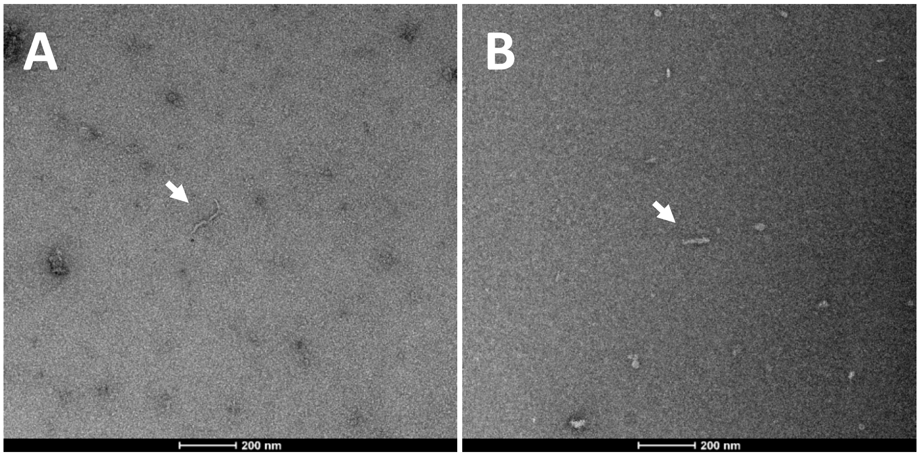

HYPER - Fraction #47 (>70 nm R_h):

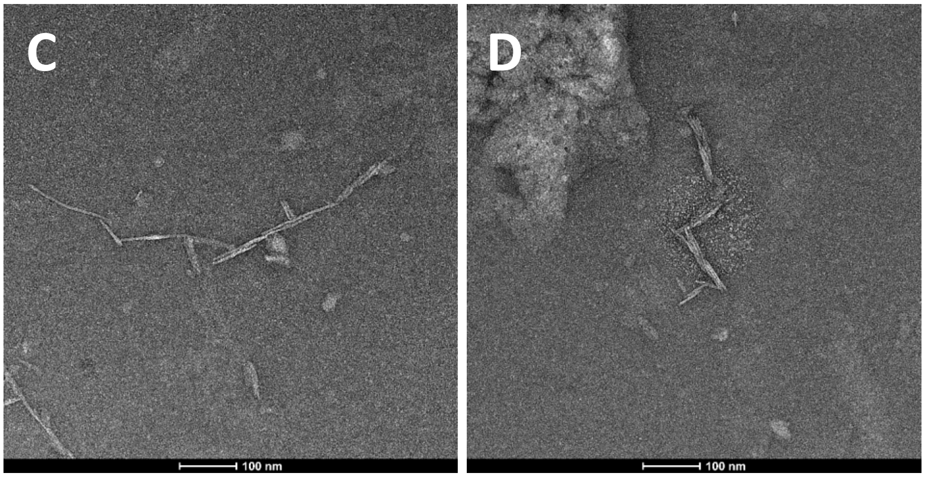

DROWSY: Fraction #30 (23 nm R_h):

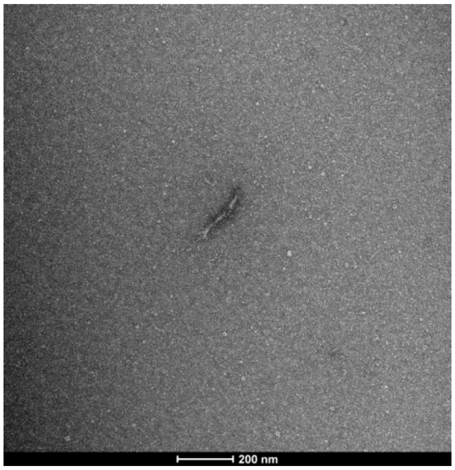

DROWSY- Faction #47 (> 70 nm R_h):

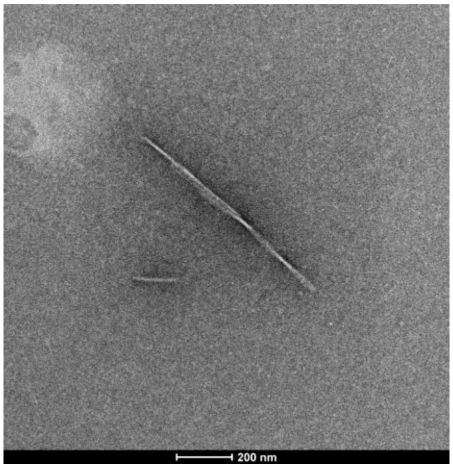

2) Light scattering data is used to fit PrP^{Sc} particles to a rod-shaped model assuming 2-protofilament fibrils and a beta-solenoid model of PrP^{Sc} structure (lines 204-209, 378-380). A model based on a parallel in-register intermolecular beta sheet (PIRIBS) structure has also been proposed for hamster 263K PrP^{Sc} [J Biol Chem 289: 24129 (2014)]. Presumably, fitting light scattering data to the PIRIBS model would lead to different particle sizes which may or may not impact some of the conclusions of the manuscript. The authors might consider briefly addressing their data in the context of the PIRIBS model and whether or not it impacts the major conclusions of their study.

We have now used the recently published PIRIBS structure for 263K fibrils solved by cryo-EM (https://doi.org/10.1101/2021.02.14.431014) and recalculated the number of PrP monomers per each PrP particle size, and the maximum possible prion particle density (see new table 1). Based on this model, we used an average diameter of 8.5nm and a contribution of 0.49 nm length per monomer.

3) For the data in Figure 7, can the authors calculate seeding activity/particle using the available data? That would allow a more direct comparison between seeding activity and particle size for 263K, Hy, and Dy hamster prions.

Although calculating seeding activity/particle could be an interesting way to compare seeding activity and particle size for the different strains, our calculation of number of particles is based on several assumptions and we cannot be sure that our lag phases are truly linear with respect to titre of seeding activity. We are therefore concerned that further mathematical linking of these numbers may heighten any error in our assumptions and be too speculative to be conclusive. We believe that the new RT-QuIC assay, in which we seed the reaction with the same volume of AF4 fractions, plus the cell infectivity assay, now provide us with enough measured data from which we can say something about seeding activity and particle size. If the reviewer strongly feels that this calculation is important, we are happy to provide it, with the above caveats clearly stated.

4) In the Conclusions (lines 434-444), the authors state that their results show that the proportion of distinctive PrP^{Sc} subpopulations determines the prion strain phenotype. In the absence of direct experimental confirmation that this is the case, “determines” is too strong a word in this context. While they can certainly draw a correlation between the properties of distinctive PrP^{Sc} populations and prion disease incubation time, the data do not support a determinative role for a specific population of PrP^{Sc} quaternary structure in dictating prion strain phenotype. The authors should consider modifying this statement.

Agreed. The sentence “Our results show that PrP^{Sc} quaternary structure is the main contributor towards PrP^{Sc} structural heterogeneity, resulting in biochemically distinctive PrP^{Sc} subpopulations, and the proportion of these subpopulations determines the prion strain phenotype” by “Our results show that PrP^{Sc} quaternary structure is **a significant** contributor towards PrP^{Sc} structural heterogeneity, resulting in biochemically distinctive PrP^{Sc} subpopulations, and the proportion of these subpopulations **correlates with** prion

strain phenotype” (L476).

5) The text on lines 201-203 refers to the PrP^{Sc} particle densities for fractions 46-50, lists what appears to be an average particle size plus and minus some undefined error with no units given, and then references Table 1. However, these data are not in Table 1. The table shows calculations for particle densities for a single brain and does not even contain particle densities for fractions 49 and 50. The authors need to be more precise about what they are discussing in this section.

The units are particles/mL. We only analyzed even numbered fractions, which is why fraction 49 is not in the table. For some brain homogenates, the largest analyzed particles eluted at fractions 46 and 48, as in the case of HY-E, but other brain homogenates, like in HY-F, eluted at fractions 48 and 50. Then we calculated PrP^{Sc} particle density average (± SD) of the fractions with the closest particles size.

Sample	Fraction #	R_h (nm)	Length (nm)	Particle density
HY-D	48	77.2	676.8	7.90E+8
HY-E	46	77.9	704.0	6.36E+8
HY-F	48	81.1	879.3	5.04E+8
Average		79	753	**6.43E+8**
SD		2	110	**1.43E+8**

Sample	Fraction #	R_h (nm)	Length (nm)	Particle density
263K-A	50	89	944	3.56E+8
263K-B	48	79	752	5.21E+8
263K-C	48	73	972	4.76E+8
Average		80	890	**4.51E+8**
SD		8	112	**8.54E+7**

Sample	Fraction #	R_h (nm)	Length (nm)	Particle density
DY-G	46	92	656	2.12E+8
DY-H	46	93	674	2.07E+8
DY-I	46	78	724	1.82E+8
Average		87	684	**2.00E+8**
SD		8	35	**1.60E+7**

6) Do lines 214-218 refer to Figure 2D? Please clarify.

The reviewer is correct. We now referenced this figure (now Fig. 2E).

7) In Figure 3, there are no data with a significance indicated by * or ** as described in the legend. Please fix to make sure that the legend and figure match.

We corrected the Fig. 3 legend.

8) Reference 20 needs to be updated to show the complete reference for the final, published manuscript.

The reference was completed.

We would like to thank you for your support and the reviewers for their excellent constructive comments, which have enabled us to improve the quality of our manuscript. It is our hope that the corrections will meet the reviewers as well as your approval and that the revised manuscript will be suitable for publication in PLOS Pathogens.

We look forward to hearing from you at your convenience.

Sincerely,

Dr Leonardo Cortez, PhD
Research Associate
Centre for Prions and Protein Folding Diseases,
Department of Medicine
University of Alberta
lcortez@ualberta.ca

Valerie Sim, MD, FRCPC,
Assistant Professor, AHFMR Clinical Investigator
Department of Medicine - Division of Neurology
University of Alberta
valerie.sim@ualberta.ca
